# Dissection and Exposure of the Whole Course of Deep Nerves in Human Head Specimens after Decalcification

**DOI:** 10.1155/2012/418650

**Published:** 2012-03-15

**Authors:** Longping Liu, Robin Arnold, Marcus Robinson

**Affiliations:** Discipline of Anatomy and Histology, University of Sydney, Anderson Stuart Building F13, Sydney, NSW 2006, Australia

## Abstract

The whole course of the chorda tympani nerve, nerve of pterygoid canal, and facial nerves and their relationships with surrounding structures are complex. After reviewing the literature, it was found that details of the whole course of these deep nerves are rarely reported and specimens displaying these nerves are rarely seen in the dissecting room, anatomical museum, or atlases. Dissections were performed on 16 decalcified human head specimens, exposing the chorda tympani and the nerve connection between the geniculate and pterygopalatine ganglia. Measurements of nerve lengths, branching distances, and ganglia size were taken. The chorda tympani is a very fine nerve (0.44 mm in diameter within the tympanic cavity) and approximately 54 mm in length. The mean length of the facial nerve from opening of internal acoustic meatus to stylomastoid foramen was 52.5 mm. The mean length of the greater petrosal nerve was 26.1 mm and nerve of the pterygoid canal was 15.1 mm.

## 1. Introduction

Topographic knowledge of the deep nerves of the head is not easily gained by students because of the deep location of these small structures and the difficulty in dissecting them out in the short amount of class time available. Anatomical drawings provide an elementary introduction to anatomy [1] but offer only theoretical knowledge.

Because dissections are unlikely to be performed by all the students [2], making good teaching prosections for gross anatomy laboratories and museums is very important.

After reviewing the literature [2–9], it was found that most studies paid attention to different points of the deep nerves of the human head without studying the whole course of the nerves. Some authors viewed parts of the deep nerves, some observed deep nerves using microscopic or CT imaging techniques. Trost et al. [2] studied the chorda tympani by marking the nerve with 3/0 iron wire in the infratemporal fossa. After meticulous repositioning of surrounding structures, standard radiography and CT scan examinations were performed. 

Just et al. [10] found that pain-related sensitivity of the ipsilateral tongue side decreases after middle ear surgery, suggesting its function influences both gustatory sensibility and intraoral trigeminal sensitivity.

Early studies [3] of the facial nerve investigated the position, course, and fibre content of its various functional components. Histological analyses of the roots, trunks and branches of facial nerve were conducted as well. All operations of this study were performed on the right side of adult animals, and not human cadavers.

Shimozawa [5] performed electron microscopic analysis of the nerve of the pterygoid canal in the mouse and found that most of the nerve fibres of the greater and deep petrosal nerve passed through into the pterygoid canal nerve, but in some specimens a few nerve fibres coursed from the deep petrosal nerve to the greater petrosal nerve or conversely.

The current study used dissection to conduct observations of the whole course of three deep nerves; the chorda tympani nerve, nerve connection between geniculate and pterygopalatine ganglia, and the facial nerve. Dissection was used to expose the whole course of the deep nerves in human heads, dissections rarely performed in gross anatomical laboratories or found in museums or atlases.

Many nerves such as the chorda tympani, nerve of the pterygoid canal, and parts of the facial nerve are hidden within the bony tissue of the human head and are difficult to dissect and expose without decalcifying the specimens first. It is very important that these nerves are demonstrated to students learning neuroanatomy [11–13]. The chorda tympani serves the taste buds in the anterior two-thirds of the tongue and carries parasympathetic and vasodilator fibres as well [7].

The aim of this study was to assess the spatial relationships of the deep nerves mentioned above, defining their exact topographical positions in order to permit surgery on these structures without damaging the neighbouring tissue. Our data will make up the lack of information in these areas.

Smith et al. [4] reported a case of a 42-year-old man with progressive right facial palsy, loss of taste on the right side of the tongue, and a mass behind the right tympanic membrane. In this case, a schwannoma was surgically removed from the vertical segment of the facial nerve, a graft was interposed, and the patient made an excellent recovery. In cases like this, our data of the facial nerve is of great value to the surgeon.

## 2. Materials and Methods

The specimens used in this study were obtained from body donors of the Discipline of Anatomy and Histology, University of Sydney. Each of the 16 temporal bone specimens was from different individuals. The age of the donors ranged from 52 to 94 with a mean age of  83. The sex of the specimens was 7 males and 9 females. Eight specimens were from the left and 8 from the right.

The half heads obtained were sectioned using a band saw to reduce the amount of tissue that needed to be decalcified prior to dissection.

There are many methods that can be used to decalcify bone tissue [14–16]. The current study used a 10% HCl solution to decalcify a total of 16 temporal bones. The specimens were immersed in 10% HCl for 7 to 10 days. Decalcification was determined to be complete once a sharp probe could be inserted into the bone without resistance. The specimens were rinsed with water to remove the acid before dissection using a scalpel and other instruments. The dissection commenced by identifying the greater petrosal nerve in the middle cranial fossa and following it posteriorly through its hiatus in the petrous temporal bone to the geniculate ganglion. The facial nerve and chorda tympani nerve were exposed after removal of the roofs of the tympanic cavity, facial canal, and internal acoustic meatus. The greater petrosal nerve was followed forward to the nerve of the pterygoid canal, then the pterygopalatine ganglion and palatine nerves were exposed.

Measurements of nerve lengths, branching distances, and ganglia size were taken using digital sliding calipers without a dissecting microscope. Fine white string was used to trace the nerves along their course, then the string was measured.

**Figure 1:**
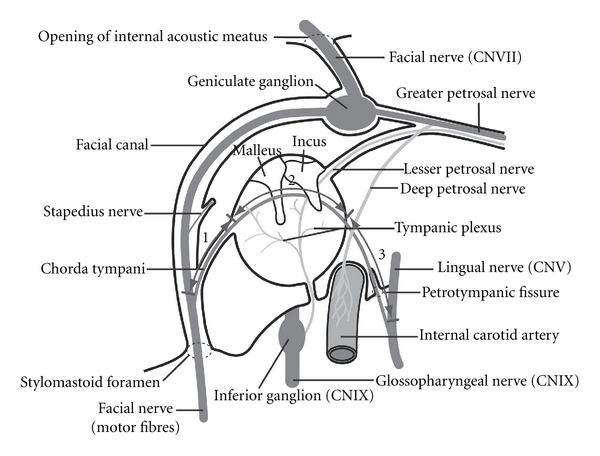
The sections of the chorda tympani between the facial nerve and lingual nerve via the tympanic cavity. Section  1: mastoid process, section 2: tympanic cavity, section 3: submandibular fossa.

The whole course of chorda tympani can be divided into three sections (Figure 1). The first section is located in the mastoid process, from the junction with the facial nerve to the tympanic cavity. The middle segment traverses the tympanic cavity, and the third section is located in submandibular fossa, extending from tympanic cavity to the junction with the lingual nerve.

Statistical analysis was performed using SPSS version 20. Sex and side differences of variables were performed using unpaired, 2-tailed *t*-tests and a significance level of 95% in all instances. Spearman's correlation analysis was conducted with all observed variables in relation to age.

**Figure 2:**
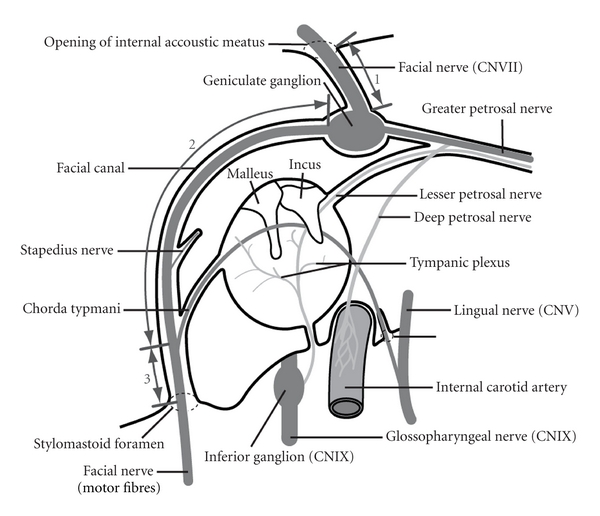
The sections of the facial nerve between the internal acoustic meatus and the stylomastoid foramen. Section  1: nerve in internal acoustic meatus, section 2: upper portion, section 3: lower portion.

The facial nerve, between the opening of internal acoustic meatus and the stylomastoid foramen, can be divided into three sections (Figure 2). The first section is from opening of internal acoustic meatus to the geniculate ganglion. The second section (upper portion) is from the geniculate ganglion to the junction of the chorda tympani the third section (lower portion) is from this junction to the stylomastoid foramen.

## 3. Results

The observed lengths of the chorda tympani nerve sections 1, 2, and 3 for each specimen are recorded in Table 1 as are the total nerve lengths calculated by adding the sections together. Likewise, the lengths of the facial nerve are recorded in Table 2.  Table 3 contains the observed dimensions of the geniculate ganglion and pterygopalatine ganglion.

### 3.1. Chorda Tympani

**Figure 3:**
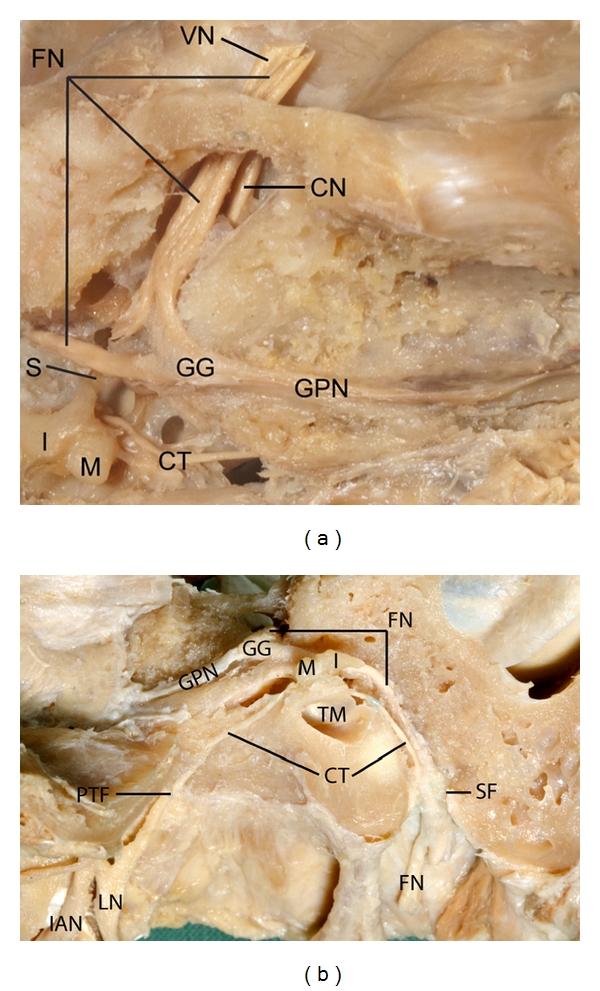
Dissection of the whole chorda tympani nerve from facial nerve, passing through the tympanic cavity and joining the lingual nerve. VN: vestibular nerve, FN: facial nerve, S: stapes, I: incus, M: malleus, CT: chorda tympani nerve, GG: geniculate ganglion, GPN: greater petrosal nerve, CN: cochlear nerve, PTF: petrotympanic fissure, LN: lingual nerve, TM: tympanic membrane, SF: stylomastoid foramen, IAN: inferior alveolar nerve.

The chorda tympani is a very fine nerve (0.44 mm in diameter within the tympanic cavity), approximately 54 mm in length connecting the facial and lingual nerves. The chorda tympani passes through the tympanic cavity in close proximity to the auditory ossicles and tympanic membrane (Figure 3). In different specimens, the chorda tympani branches from the facial nerve in varying locations.

The distance between the chorda tympani and stylomastoid foramen ranges from 5.93 mm to 21.63 mm, the average distance being 13.32 mm. The lingual nerve length, between foramen ovale and chorda tympani, ranges from 14.79 mm to 36.46 mm, the average being 21.59 mm.

### 3.2. The Facial Nerve

The whole facial nerve was exposed in each half head. The mean length of the facial nerve in the internal acoustic meatus and facial canal was found to be 52.50 mm based on 16 specimens. The mean length of the facial nerve in the internal acoustic meatus was 15.93 mm. The mean length of the upper portion of facial nerve (in the facial canal, above chorda tympani) was 23.25 mm and the lower portion of facial nerve (below chorda tympani) the mean length was 13.33 mm.

### 3.3. The Greater Petrosal, Palatine Nerves and the Nerve of the Pterygoid Canal

**Figure 4:**
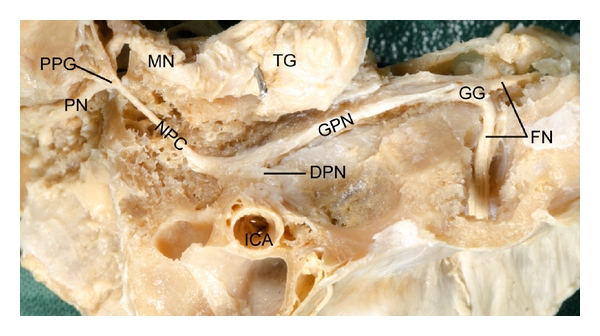
Dissection of the nerve connections between the geniculate and pterygopalatine gangia. PN: palatine nerves, PPG: pterygopalatine ganglion, MN: maxillary nerve, NPC: nerve of pterygoid canal, DPN: deep petrosal nerve, TG: trigeminal ganglion, ICA: internal carotid artery, GPN: greater petrosal nerve, GG: geniculate ganglion, FN: facial nerve.

After delicate dissection, the facial nerve, greater petrosal nerve, palatine nerves, and the nerve of the pterygoid canal were exposed in each of 13 temporal bones. The mean lengths of the following nerves were: greater petrosal nerve 26.05 mm, nerve of the pterygoid canal 15.10 mm, greater palatine nerve 26.18 mm and lesser palatine nerve 26.91 mm. The pterygopalatine ganglion is located in the pterygopalatine fossa and close to the posterior wall of the maxillary sinus (Figure 4). The pterygopalatine ganglion is pyramidal in shape and measures approximately 2.82 × 2.82 × 1.75 mm.

Considering all the observed measurements, sex and side differences were analysed using 2-tailed, unpaired *t*-tests. No significant differences were found with relation to sex or side. Spearman's correlation analysis found that age positively correlated with the total length of the chorda tympani nerve (*P* = 0.041, *r* = 0.515) and nerve of the pterygoid canal (*P* = 0.037, *r* = 0.583). Although significant statistically, the correlations themselves are weak and may be due to the small sample size.

## 4. Discussion

The chorda tympani nerve arises from the facial nerve above the stylomastoid foramen; it runs superiorly and anteriorly through the posterior wall of the tympanic cavity and then into the cavity. It is situated close to the medial surface of the tympanic membrane and crosses the handle of the malleus. The nerve passes through the petrotympanic fissure and runs inferiorly and anteriorly deep to the lateral pterygoid muscle, is crossed by the middle meningeal artery, and finally joins the posterior border of the lingual nerve at an acute angle.

The chorda tympani nerve conducts the gustatory fibres for the anterior two-thirds of the tongue and the parasympathetic fibres to the submandibular and sublingual glands.

Gray's Anatomy [17] describes the chorda tympani nerve arising from the facial nerve about 6 mm above the stylomastoid foramen. Trost et al. [2] describe the chorda tympani emerging from the third intraosseous segment, 2 or 3 cm above the stylomastoid foramen but only made measurements of the chorda tympani in the infratemporal fossa region. According to our observations, the lower portion of the facial nerve (from junction of the chorda tympani to stylomastoid foramen) is 13.33 mm in length. Our measurements are intermediate between those found by Davies and Coupland [17] and Trost et al. [2].

Dobozi [18] measured the geniculate ganglion from histological sections of the temporal bone. He found that in the horizontal plane the ganglion is triangular in shape and has an average length of 1.09 mm; the average width of this structure is 0.76 mm with an average height of 0.6 to 0.8 mm. These observations differ from the current study, possibly because we measured the whole geniculate ganglion with the unaided eye.

Conditions such as Bell's palsy and Ramsay Hunt syndrome (geniculate neuralgia or otic neuralgia) are caused by disorders of the facial nerve and its branches between the geniculate and pterygopalatine ganglia. Treatment of crocodile tears, chronic vasomotor rhinitis, and allergic rhinitis by neurectomy of the vidian nerves [8, 19] requires specific anatomical knowledge of this region. Nomura [19] introduced a new surgical technique for vidian neurectomy. Using Caldwell-Luc's procedure, he opened the maxillary sinus, and thus performed a transantral subperiosteal vidian neurectomy. Our data and specimens show the close relationship between the posterior wall of the maxillary sinus and the pterygopalatine ganglion and pterygoid canal. 

Concerning the greater petrosal nerve (GPN) and nerve of pterygoid canal, Tubbs and colleagues [9] state that the GPN runs for an average of 11 mm in the middle cranial fossa (range: 7–13 mm), medial to the lesser petrosal nerve (LPN). However, our observations show that the average total length of the GPN is 26.05 mm. We observed that the nerve of the pterygoid canal ranges in length from 12.5 to 18.5 mm. The mean length of the nerve of the pterygoid canal is 15.1 mm. Tubbs and colleagues [9] reported that the vidian nerve (nerve of the pterygoid canal) ranges in length from 10 to 12 mm, differing somewhat to our observations.

The facial nerve controls expression on the face, tearing, taste, and even hearing to some extent. Many diseases can cause a facial nerve disorder, such as Bell's palsy. These diseases include ear infection, trauma, tumors of the ear or brain, stroke, and genetic disease [4]. The whole course of facial nerve is rarely found in anatomical museums and atlases because it is difficult to expose. Our specimens, showing the deep nerves and whole course of facial nerve, will enhance the quality of neuroanatomy teaching and supplement anatomical atlases and museums. Kudo and Nori [6] did some research on topography of the facial nerve in the human temporal bone, but they did not expose the whole course of facial nerve in human head specimens.

Based on our experience, we give the following suggestions for guidance of how to dissect and expose the deep nerves of the head region after decalcification to help reduce potential damage to the nerves.

(1) The dissector should have good knowledge about the deep nerves of head before dissecting the specimens. Dissectors need to learn the related anatomic knowledge before beginning the dissection.

(2) As mentioned above, the roof of the tympanic cavity is the best region for starting the dissection. After removing the roof of the tympanic cavity, the geniculate ganglion, greater petrosal nerve, and chord tympani nerve can be easily found. 

(3) Some deep nerves in the head are very small, located entirely in the bone tissue and can be easily destroyed during dissection. Care is needed at all times.

(4) A tracing method can be used to track the deep nerves. Tracing the chorda tympani nerve from tympanic cavity and petrotympanic fissure, removing the bone tissue little by little. To expose the pterygopalatine ganglion and the nerve of the pterygoid canal, first find the greater and lesser palatine nerves, 10 mm anterior to the orifice of the auditory tube. Resect the bone tissue, then dissect superiorly to find the ganglion. Take the bone tissue off in a posterior direction and try to find the nerve of the pterygoid canal.

## 5. Conclusions

The course of the facial nerve and all nerves mentioned above is complex and located deep within the bones of the skull. Decalcification of the bone is necessary before dissection can begin. There is sometimes concern that decalcification will damage the other tissues such as nerves, blood vessels, or muscles. There is no risk to these structures. A rapid decalcification method is sometimes used in order to preserve ultrastructure of temporal bones using a microwave [15]. Using the method described in this paper, we did not find any destruction of those tissues after 10 days in 10% HCl.

Perfect decalcification is the key success of our experiment. The larger the tissue block, the more time is needed decalcifying the tissue. If decalcification time is insufficient it may be extended. If the concentration of HCl is below 10%, more time is needed.

Sound experience and dissection skill are necessary to make delicate dissections of head specimens exposing the deep nerves within bony tissue. The author (L. Liu) has 20 years of experience dissecting human head specimens. Practice dissecting the region of interest is also conducive to excellent results.

The best specimens for exposing facial nerve are half heads with the falx cerebri retained because the brain and intracranial portion of the facial nerve are secured and protected. Specimens showing the whole course of facial nerve will enhance the quality of teaching and supplement anatomical atlases and museums.

## Figures and Tables

**Table 1:** Measurements of the chorda tympani nerve in millimetres.

Cadaver identification number	Section of chorda tympani nerve			Total length
	Section 1 Mastoid process	Section 2 Tympanic cavity	Section 3 Submandibular fossa	
875604	11.95	13.00	29.12	54.07
876302	9.93	9.04	33.26	52.23
876506	4.54	9.97	30.13	44.64
881501	11.65	9.04	33.30	53.99
8802	5.71	8.01	39.27	52.99
8823	8.99	9.87	39.54	58.40
8274	14.12	9.34	42.90	66.36
882401	13.38	9.62	29.19	52.19
8803	12.73	9.43	52.42	74.58
8697	2.79	11.27	28.87	42.93
8750	6.23	9.56	35.84	51.63
881601	14.18	7.86	32.93	54.97
8814	6.16	11.16	35.84	53.16
8849	6.21	10.14	36.09	52.44
8810	9.26	10.34	30.37	49.97
8850	6.29	9.68	40.31	56.28

Mean	9.01	9.83	35.59	54.43
SD	3.68	1.24	6.28	7.49

**Table 2:** Measurements of the facial nerve in millimetres.

Cadaver identification number	Portion of facial nerve			Facial nerve total length
	Facial nerve in internal acoustic meatus	Upper portion above chorda tympani	Lower portion below chorda tympani	
875604	13.52	27.90	5.93	47.35
876302	16.23	21.73	12.62	50.58
876506	17.55	16.44	21.63	55.62
881501	16.26	27.74	15.91	59.91
8802	13.15	16.60	17.12	46.87
8823	15.40	22.83	13.06	51.29
8274	14.75	26.88	8.64	50.27
882401	17.58	23.40	6.36	47.34
8803	17.76	29.92	11.56	59.24
8697	17.14	18.53	16.59	52.26
8750	12.61	24.40	13.87	50.88
881601	18.19	29.31	9.84	57.34
8814	19.17	21.47	12.96	53.60
8849	13.71	19.51	15.94	49.16
8810	15.23	23.56	13.91	52.70
8850	16.65	21.70	17.26	55.61

Mean	15.93	23.25	13.33	52.50
SD	1.97	4.26	4.20	4.11

**Table 3:** Measurements of the geniculate and pterygopalatine ganglia in millimetres.

Cadaver identification number	Geniculate ganglion		Pterygopalatine ganglion		
	Length	Width	Length	Height	Width
881501	2.15	2.23	2.84	2.42	2.40
8802	1.58	1.78	3.41	3.21	1.47
8823	2.45	2.03	2.79	3.37	2.09
8274	2.16	2.09	2.50	2.73	1.77
882401	2.27	1.90	2.28	2.57	1.39
8803	2.37	2.05	3.37	3.55	2.37
8697	1.94	2.00	1.74	2.20	1.33
8750	2.05	1.77	2.67	2.55	1.05
881601	2.35	1.92	3.54	3.33	1.61
8814	2.13	1.70	3.19	3.03	1.42
8849	2.36	2.22	3.11	2.55	2.00
8810	1.98	1.73	1.93	2.05	1.35
8850	2.08	1.82	3.26	3.14	2.45

Mean	2.14	1.94	2.82	2.82	1.75
SD	0.23	0.18	0.57	0.48	0.47
